# Multiomic elucidation of a coding 99-mer repeat-expansion skeletal muscle disease

**DOI:** 10.1007/s00401-020-02164-4

**Published:** 2020-05-25

**Authors:** Alessandra Ruggieri, Sergey Naumenko, Martin A. Smith, Eliana Iannibelli, Flavia Blasevich, Cinzia Bragato, Sara Gibertini, Kirston Barton, Matthias Vorgerd, Katrin Marcus, Peixiang Wang, Lorenzo Maggi, Renato Mantegazza, James J. Dowling, Rudolf A. Kley, Marina Mora, Berge A. Minassian

**Affiliations:** 1 Department of Neuroimmunology and Neuromuscular Diseases, Fondazione IRCCS Neurological Institute Carlo Besta, Milan, Italy; 2 Department of Molecular and Translation Medicine, Unit of Biology and Genetics, University of Brescia, Brescia, Italy; 3 Centre for Computational Medicine, Hospital for Sick Children, Toronto, ON Canada; 4 CHU Sainte-Justine Research Center, Montreal, QC Canada; 5 Department of Biochemistry and Molecular Medicine, Faculty of Medicine, Université de Montréal, Montreal, QC Canada; 6 St-Vincent’s Clinical School, Faculty of Medicine, UNSW Sydney, Sydney, Australia; 7 Garvan Institute for Medical Research, Darlinghurst, NSW Australia; 8 PhD Program in Neuroscience, University of Milano-Bicocca, Monza, Italy; 9 Department of Neurology, Heimer Institute for Muscle Research, University Hospital Bergmannsheil, Ruhr-University Bochum, Bochum, Germany; 10 Medizinisches Proteom-Center, Ruhr-University Bochum, Bochum, Germany; 11 Program in Genetics and Genome Biology, Hospital for Sick Children Research Institute, Toronto, ON Canada; 12 Department of Neurology and Clinical Neurophysiology, St. Marien-Hospital Borken, Klinikum Westmuensterland, Borken, Germany; 13 Division of Neurology Department of Pediatrics, University of Texas Southwestern, Dallas, TX USA

Twenty-two individuals across four generations suffer a chromosome 19p13.3-linked autosomal dominant progressive myopathy with distinctive pathology including rimmed ubiquitin-positive autophagic vacuolation [6] (Fig. 1 and Supplementary data). A recombination in the newest (youngest) affected patient (V:13) and repeat linkage analysis on six patients (IV:10, IV:17, III:18, IV:3, IV:23, V:13) refined the disease haplotype to 5.12 Mb containing 164 genes. Sanger (24 genes), whole-exome, whole-genome (Supplementary Table 1) and whole skeletal muscle RNA sequencing proved unrevealing.

**Fig. 1:**
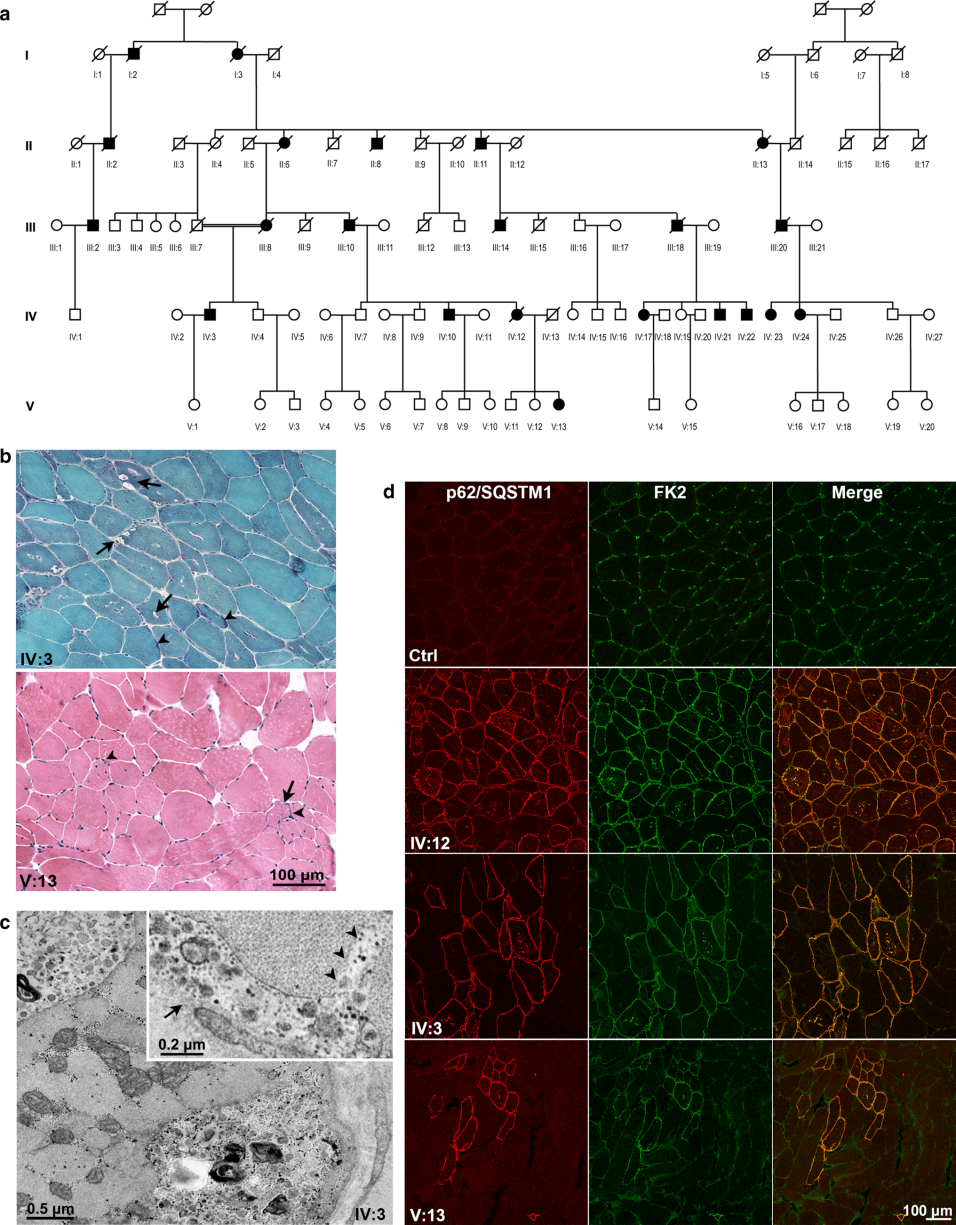
Pedigree and muscle histopathology. **a** Pedigree. **b** Gomori trichrome and H&E staining, respectively, in patients IV:3 and V:3, showing vacuoles (arrows) rimmed, empty, or containing granular or basophilic material (arrowheads) mainly located in the subsarcolemmal region of fibers, fiber size variability, central nuclei and mildly increased endomysial spaces. Note minimal changes in V:13. **c** Electron micrographs unveiling (upper panel) granular debris within a small subsarcolemmal vacuole (arrow) opening to the fiber’s surface and sarcolemmal interruption (arrowheads); (lower panel) vacuoles located in the subsarcolemmal region or deep in the sarcoplasm, containing small vesicles, membranous bodies and granular debris. **d** Confocal microscopy of p62/SQSTM1 and FK2 immunostaining showing positivity and almost complete overlap of both proteins in vacuoles and subsarcolemmae of affected fibers, more numerous with increasing clinical severity

Immunohistochemical workup showed that patients’ vacuoles and subsarcolemmal regions stained positive for FK2 and p62/SQSTM1 markers, respectively, of ubiquitinated proteins and autophagy (Fig. 1d). The number of stained fibers correlated with clinical severity (Supplementary data). To query the FK2 target(s), we microdissected vacuoles and unaffected myofiber parts for quantitative mass spectrometry. Among the more than 700 identified proteins (Supplementary Table 2), perilipin-4 was the most highly (almost 20-fold) over-represented in vacuoles versus control myofiber regions (Supplementary Fig. 2). Perilipins coat the phospholipid monolayer surrounding lipid droplets and regulate the latter [2]. All five perilipins share an amphipathic domain composed of an 11-amino acid (aa) sequence, which is particularly extensive in perilipin-4, the 11-mer being repeated three times to generate a 33-mer, in turn repeated 29 or 31 times [11] (Supplementary Fig. 3). Perilipin-4 is the most abundantly expressed perilipin in muscle, notwithstanding which, aside from reduced cardiac triacylglycerol levels, its absence in mouse results in no cardiac, skeletal muscle or other impairment [10, 11]_._

**Fig. 2:**
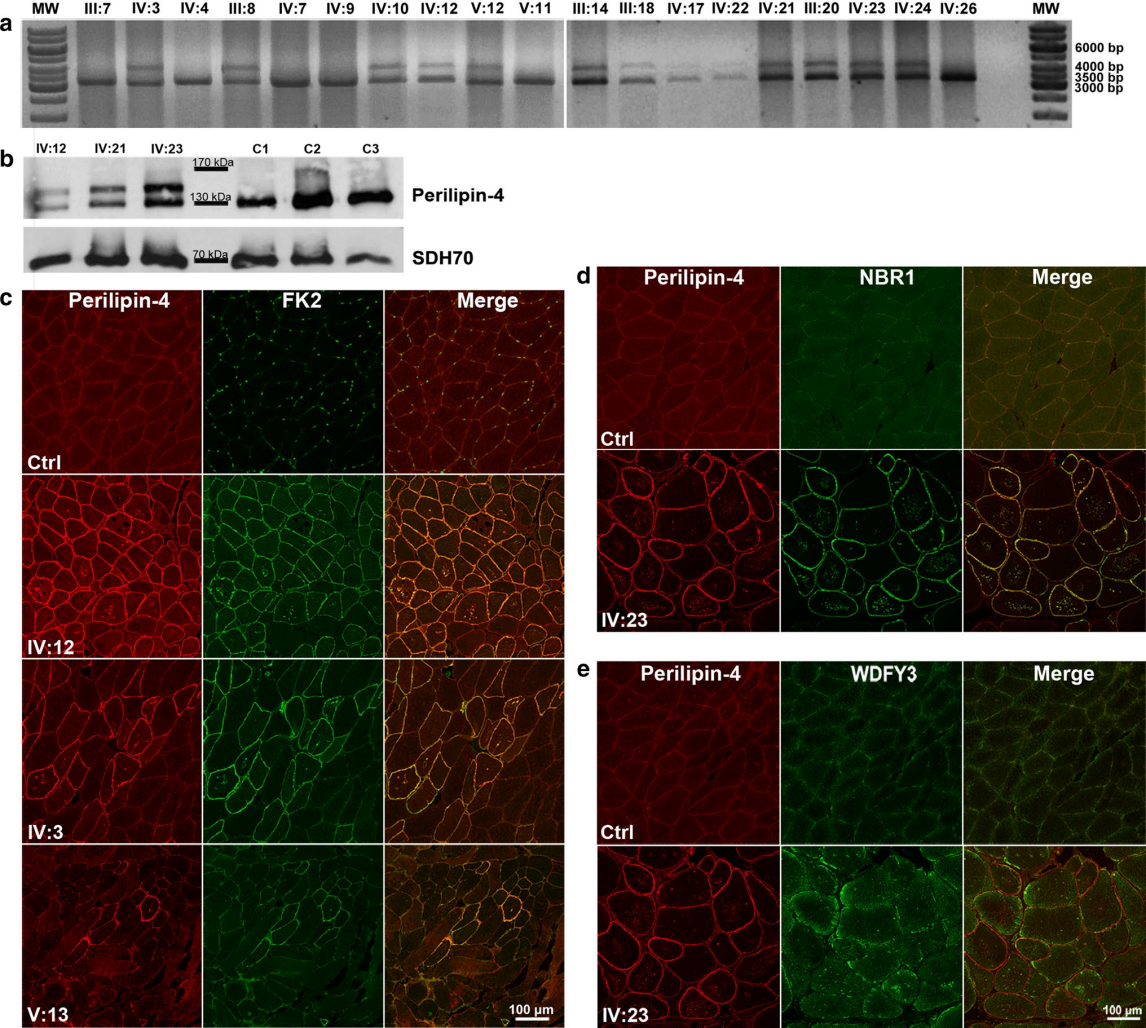
Perilipin-4 expression and aggrephagy. **a** Exon 3 PCR amplification showing besides the wild-type band, a second one approximately 1000 bp higher only in affected family members. **b** Perilipin-4 western blot revealing a second band in patient muscle, absent in controls. **c** Immunohistochemistry showing perilipin-4 within vacuoles and in the subsarcolemmal region in all affected fibers, overlapping with FK2 by confocal microscopy analysis. **d**, **e** Co-localization of perilipin-4 and aggrephagy-related proteins NBR1 (**d**) and WDFY3 (**e**), showing upregulation of both in patient muscle, with good overlap of NBR1-perilipin-4and increase of WDFY3 near perilipin-4

Since *PLIN4* maps to our linked region, we revisited the genomic and transcriptomic patient data and noticed an unusually high coverage in *PLIN4* exon 3 (Supplementary Fig. 4). PCR amplification of this exon in patient genomic DNA and muscle RNA revealed the wild-type band, and a second ~ 1000 bp higher band (Fig. 2a) not present in unaffected relatives or in 60 ethnic controls. The 31 × 33-aa amphipathic domain of perilipin-4 is encoded by 31 × 99 repetitive sequences in exon 3 [4], which poses a computational challenge for aligning short sequencing reads. We amplified cDNA from patient muscle RNA and obtained Oxford Nanopore long-read sequencing, which confirmed that the higher band is an expansion of the normal 31 × 99-nucleotide sequence to 40 × 99 bases, resulting in 297 (9 × 33) extra amino acids (Supplementary Fig. 5). Muscle extract Western blotting with a perilipin-4 antibody showed the presence of a second band consistent in size with the genetic expansion in patients, and absent from controls (Fig. 2b). Immunohistochemistry with the same antibody showed a major increase in perilipin-4 positivity in subsarcolemmal regions and vacuoles of patients compared to controls. The perilipin-4 signal most exactly reproduced the staining with the FK2 (Fig. 2c) and p62/SQSTM1 antibodies (Supplementary Fig. 6). These staining correlated with the diseased muscle fiber type, namely, slow-twitch Type I fibers, known to contain the highest amounts of intramyocellular lipids. Oil Red O staining showed normal lipid content and distribution (Supplementary Fig. 7 and data). Aggrephagy pathway components beyond FK2 and p62/SQSTM1, namely, NBR1 and WDFY3, were upregulated, the former (Fig. 2d) colocalizing with perilipin-4, FK2 and p62/SQSTM1, the latter (Fig. 2e) increased in subsarcolemmae near perilipin-4 positivity but without co-localization. In aggrephagy, p62/SQSTM1 interacts with NBR1, and the two, as an autophagy receptor complex and through their shared LC3-interacting regions, bridge the aggregating ubiquitinated proteins with LC3. Meanwhile, WDFY3 shuttles from the nucleus to the cytoplasm to scaffold the overall structure with PtdIns3P-containing membranes and encapsulate the aggregates in autophagosomes for degradation [8]. The present disease is characterized by dominantly inherited progressively increasing mobilization of aggrephagy at sites of progressive accumulation of a mutated protein, suggesting that the mutation is leading to aggregation, likely through misfolding, exceeding aggrephagic capacity. Continuous formation and fusion of failing aggrephagic vesicles possibly leads to ever larger vacuoles, which disrupt the organization of myofibers and alter their contractile abilities, resulting in atrophy.

Many cases of Inclusion Body Myopathy, the most common of the myopathies, exhibit aggrephagic activation, including NBR1 deposition, not dissimilar to the present patients [5]. The proportion of cases that are due to misfolded proteins, potentially including perilipin-4, remains to be determined.

Perilipin-4 shares its amphipathic domain structure with α-synuclein and exchangeable lipoproteins (ApoA, ApoC and ApoE) [3]. All known mutations (all missense) of α-synuclein and ApoA1 in familial Parkinson disease and amyloidosis, respectively, localize to these proteins’ amphipathic regions and transition the repeating helices of these domains to amyloidogenic β pleats [1, 7]. Genomic repeat sequences predispose to expansion [9]. To our knowledge, ours is the first report of an amphipathic domain repeat expansion in disease, and identification of the expansion was only possible with long-read sequencing. The possible occurrence of germline or somatic pathogenic amphipathic region repeat expansions in proteins possessing these domains in their related diseases should be explored.

## Electronic supplementary material

Below is the link to the electronic supplementary material.

Supplementary file1 (PDF 2080 kb)

Supplementary file2 (XLSX 60 kb)

Supplementary file3 (XLSX 474 kb)
