# Supplementary material for: Multiomic elucidation of a coding 99-mer repeat-expansion skeletal muscle disease
Source: Acta Neuropathol. 2020 May 25;140(2):231–5. doi: 10.1007/s00401-020-02164-4 (PMC7360652; doi:10.1007/s00401-020-02164-4)
Supplement: Supplementary file 1 — Supplementary file1 (PDF 2080 kb) [file 401_2020_2164_MOESM1_ESM.pdf]

**SUPPLEMENTARY MATERIAL Acta Neuropathologica**

# Multiomic elucidation of a coding 99-mer repeat-expansion skeletal muscle disease

**Alessandra Ruggieri, Sergey Naumenko, Martin A. Smith, Eliana Iannibelli, Flavia Blasevich, Cinzia Bragato, Sara Gibertini, Kirston Barton, Matthias Vorgerd, Katrin Marcus, Peixiang Wang, Lorenzo Maggi, Renato Mantegazza, James J. Dowling, Rudolf A. Kley, Marina Mora, Berge A. Minassian**

**Correspondence**:

Berge A. Minassian
berge.minassian@UTSouthwestern.edu

Alessandra Ruggieri
alessandra.ruggieri@istituto-besta.it

## SUPPLEMENTARY DATA

### Morphological findings

Pathological features of muscle were: markedly variable fiber size with hypertrophic and angulated atrophic fibers, mild to moderate increase in endomysial and perimysial fat and fibrous connective tissue, central nuclei, fiber splitting, and vacuoles. Vacuoles were often rimmed and appeared either empty or filled with basophilic and/or granular material. They were present either at the inner of the fibers where their size was variable, sometimes replacing most of the sarcoplasm; or, more often, they were clustered, multiple and small in size, at the fiber surface (Fig. 1b and Supplementary Fig. 1a).

The severity of the histopathologic changes varied between patients and correlated with disease severity (Fig.1b and Supplementary Fig.1a and 2a). In particular a muscle biopsy in the youngest affected case (V:13) showed minimal changes such as variation in fiber size, a few central nuclei, and basophilic underlining of the surface in rare fibers (Fig. 1b).

The electron microscopy analysis revealed that vacuoles were often membrane-limited, and, especially those at the fiber surface, were also bordered by the basal lamina. Vacuoles contained numerous membranous bodies, partially degraded organelles, and amorphous or granular material (Supplementary Fig. 1b,c,d,f). They were located either within the cytoplasm where they were usually large in size, and at the fiber surface, where they were smaller in size and either single or multiple, often abutting the extracellular space (Supplementary Fig. 1b,c,f) (see also previous Di Blasi et al.'s report [3]). These peripheral multiple microvacuoles likely correspond to the subsarcolemmal regions positive for Perilipin-4, ubiquitinated proteins and autophagic markers seen in light microscopy. The basal lamina, in addition to limiting many vacuole surfaces, often formed multiple layers and loops in the extracellular space facing the vacuoles (Supplementary Fig.1b,c). Glycogen was abundant in the intermyofibrillar spaces and close to and within vacuoles. Tubulo-filamentous inclusions were not observed in the sarcoplasm or in nuclei, nor myofibrillar, or mitochondrial alterations. Sarcoplasmic reticulum cisternae appeared sometimes enlarged especially at the triads (Supplementary Fig. 1b,f); lipid droplets were rare, appearing normally empty or, in a few cases, with a dense core (Supplementary Fig. 1b).

Percentages of fibers with increased positivity to perilipin-4 seemed to be well correlating with the patients' phenotype severity measured according to the Walton and Gardner-Medwin scale, as shown in the Supplementary Fig. 2a graph which only included those patients for whom neurological examination was performed at time of biopsy.

The number of perilipin-4- or FK2-positve fibers directly correlated with clinical severity, ranging from very few in patients IV:17 and V:13, both practically asymptomatic, to all fibers in the most severely affected patients (Supplementary Fig.2).

Perilipin-4 immunostaining in muscle biopsies from genetically determined vacuolar myopathies such as DNAJB6-, GNE-, VMA21-, LAMP2-, or GAA-mutated myopathies showed lack of positivity in the vacuoles and in the subsarcolemmal region of the fibers, except in a few spots due to autofluorescence (Supplementary Fig. 2),

Quantitation of co-localization between perilipin-4 and NBR1, performed in patient IV:3, revealed a significant increase of co-localization signal compared to a control (a VCP-mutated patient with autophagic vacuoles) (IV:3 = 0.7427 ± 0.02107, vs Ctrl = 0.3130 ± 0.02170, p=0.0001). Similarly, perilipin-4 and FK2 co-localization signal (IV:3 = 0.7473 ± 0.01617, vs Ctrl: = 0.2803 ± 0.001333, p<0.0001), and perilipin-4 and p62 co-localization signal (IV:3 = 0.7070 ± 0.00493, vs Ctrl = 0.2923 ± 0.03308, p=0,0002) were significantly increased in the patient.

# SUPPLEMENTARY FIGURES

**Supplementary Fig. 1** Muscle morphological features

(a) Gomori trichrome staining of patient IV:21 muscle showing presence of numerous vacuoles at the fiber surfaces which appear rimmed and filled with granular basophilic material (arrows),

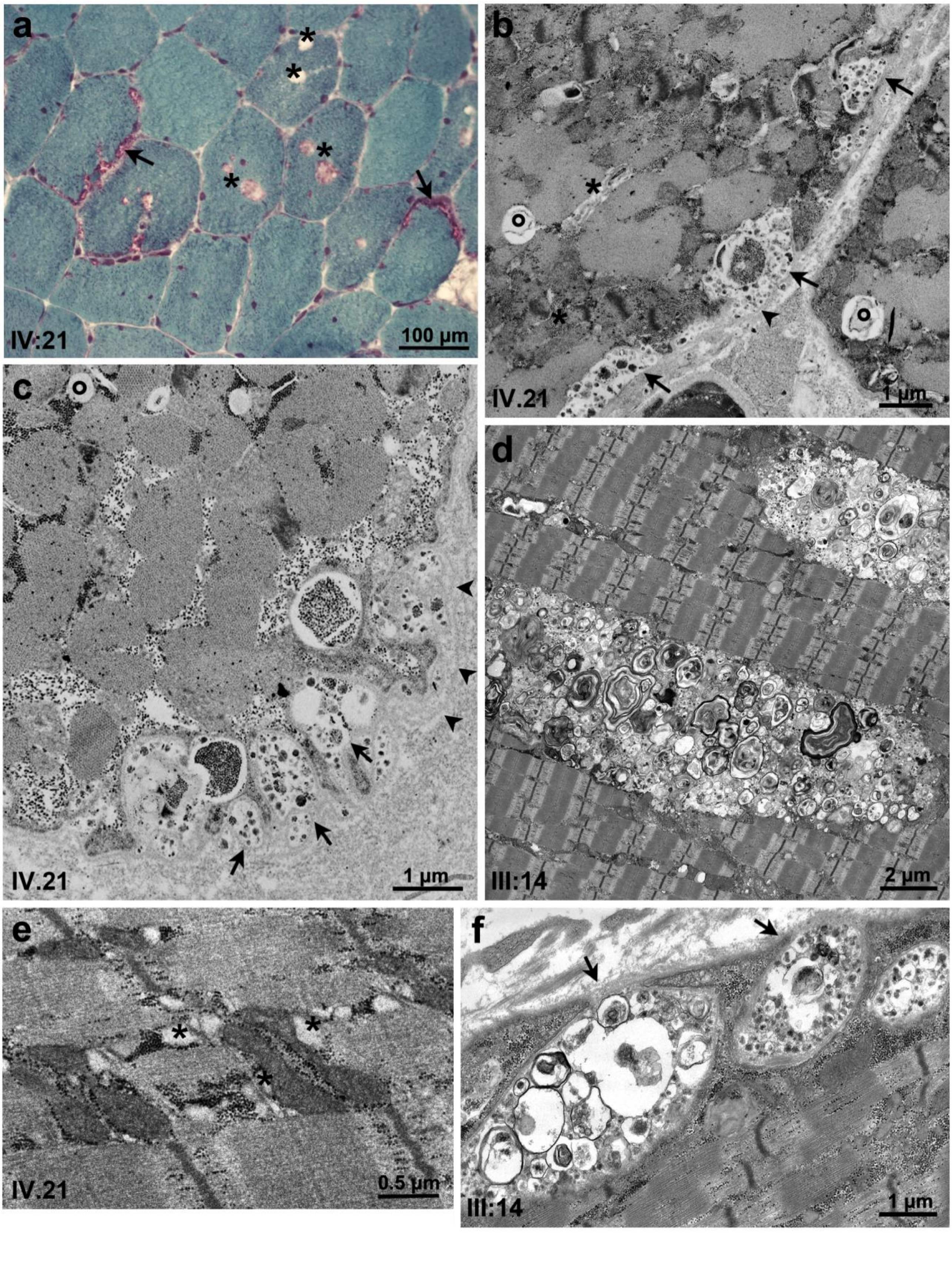

and centrally located vacuoles empty or containing slightly basophilic material (asterisks). (b-f) Electron microscopy images of transverse and longitudinal ultrathin muscle sections showing: cytoplasmic and peripheral vacuoles sometimes membrane-limited, filled with membranous bodies, partially degraded organelles, and amorphous or granular material (b,c,d,f); peripheral multiple vacuoles which are small in size and often abutting the extracellular space (b,c). Some of these vacuoles are partially or totally delimited only by the basal lamina (arrows) that also forms multiple layers and proliferating loops in the extracellular space facing the vacuoles (arrowheads). (b,c,d,f) Glycogen particles are present in the intermyofibrillar spaces and within vacuoles; (b,f) sarcoplasmic reticulum cisternae and triads appear enlarged (asterisks); (b,c) lipid droplets are rare, and sometimes with a dense core (circles).

**Supplementary Fig 2** Quantitation of perlipin-4-positive fibers and of its co-localization with NBR1, FK2, p62, and perilipin-4 immunostaining of in genetically determined vacuolar myopathies

(a) Graph showing quantitation of perilipin-4-positive fibers relative to clinical severity, evaluated according to the Walton and Gardner-Medwin scale, at the time of muscle biopsy. For each patient the scale's values are reported. (b) quantitation of perilipin-4 signal co-localization with NBR1, FK2 and p62, showing significant increased colocalization in patient IV:3 compared to a control. Comparisons between the two groups were done by two-tailed unpaired t-test. *** indicates p≤0.0002 (c) Perilipn-4 immunostaining in muscle from genetically determined vacuolar myopathies showing lack of positivity in vacuoles and normal low positivity in subsarcolemmal regions of the fibers. The few spots apparently postitive in LAMP2- and GAA-mutated patient muscles are autofluorescent lysosomal corpuscles.

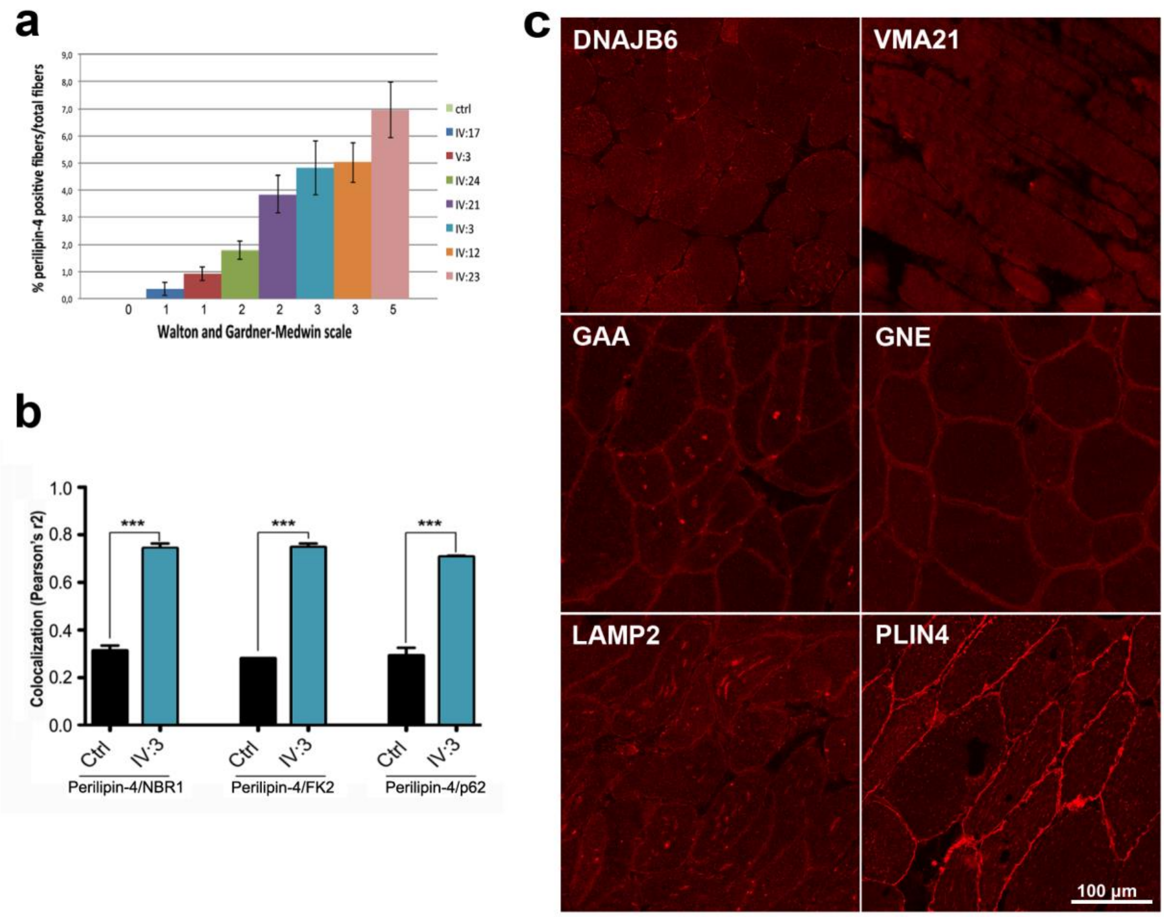

**Supplementary Fig 3** Laser microdissection proteomic analysis

(a) Representative images showing how equivalent portions of muscle fibers, either containing rimmed vacuoles (labeled VAC), or the sarcoplasm of the same vacuolated fiber (PCI, patient control I), or a portion of a healthy-looking fiber (PCII), or of a healthy control muscle fiber (HC) were collected by laser micro-dissection for proteomic analysis. (b) Relative ratios of perilipin-4 content in the various portions of the fibers, after proteomic analysis.

a

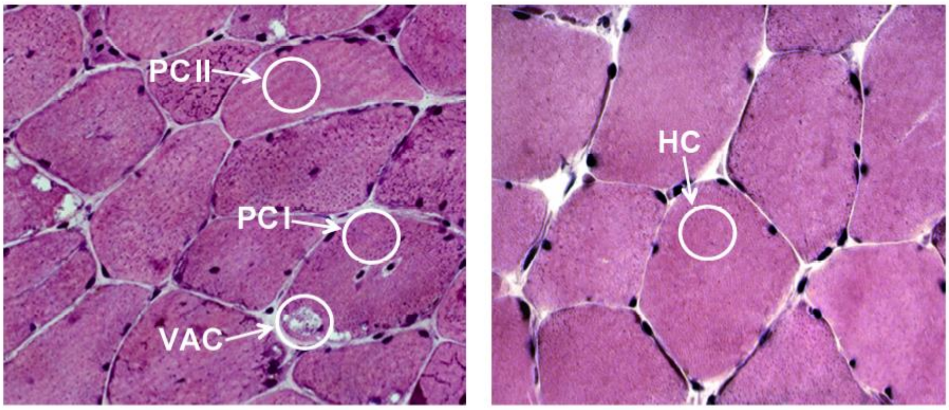


b

| | | |
|---|---|---|
| Accession | Locus | Ratio VAC / HC |
| Q96Q06 Perilipin-4 | chr19:4502192-4517716 | 19,57080292 |
| Accession | Locus | Ratio VAC / PC II |
| Q96Q06 Perilipin-4 | chr19:4502192-4517716 | 19,57080292 |
| Accession | Locus | Ratio VAC / PC I |
| Q96Q06 Perilipin-4 | chr19:4502192-4517716 | 0,850904475 |
| Accession | Locus | Ratio PC I / PC II |
| Q96Q06 Perilipin-4 | chr19:4502192-4517716 | 23 |

**Supplementary Fig. 4** Schematic representation of perilipins

As mentioned in the text, the perilipin-4 33-mer repeat sequence has a normal variation in the human population of 29 or 31 repeats (https://www.uniprot.org/uniprot/Q96Q06)

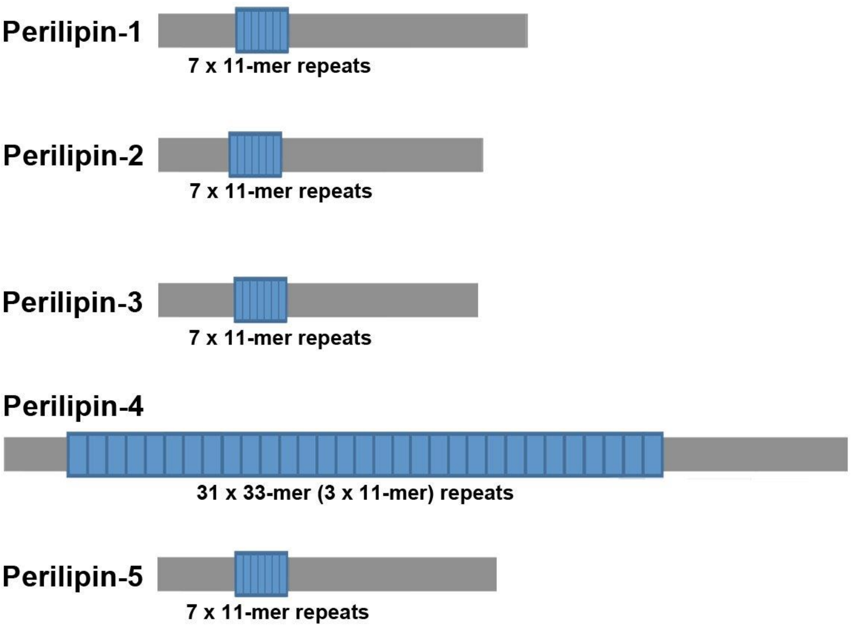

**Supplementary Fig. 5** Next generation sequencing

IGV (Integrative Genomics Viewer) [9] visualization of next generation sequencing results, both RNA (rows 1, 2) and genome (rows 3-6) views showing presence in patients of a high peak of coverage (rows 1-4) in PLIN4 exon 3 not present in the healthy siblings (row 5, 6).

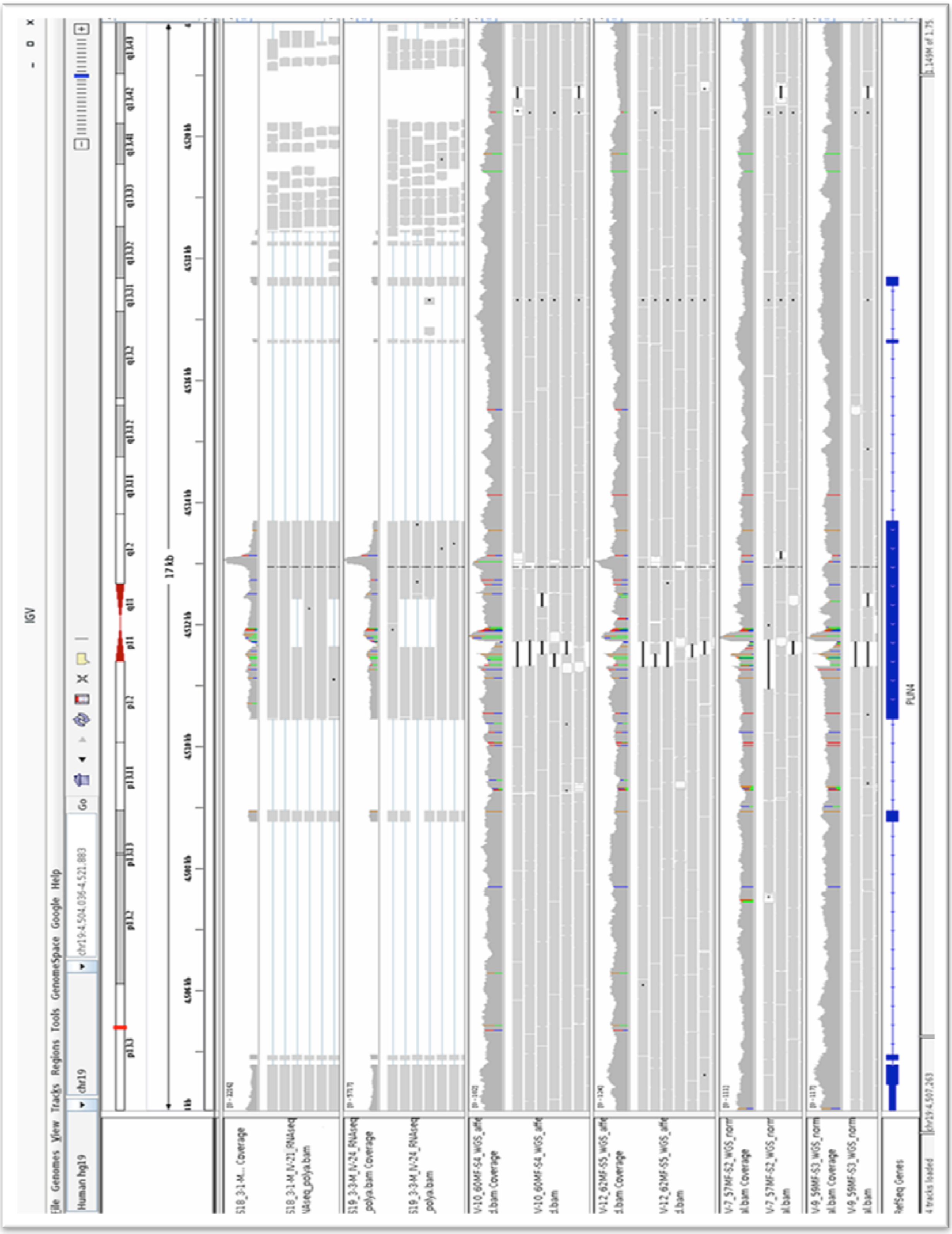

**Supplementary Fig. 6** Perilipin-4 structure

Schematic representation of the 33-mer wild-type amphipathic region and its expansion in the mutant protein. The 33-mer helices were sketched using Heliquest [4].

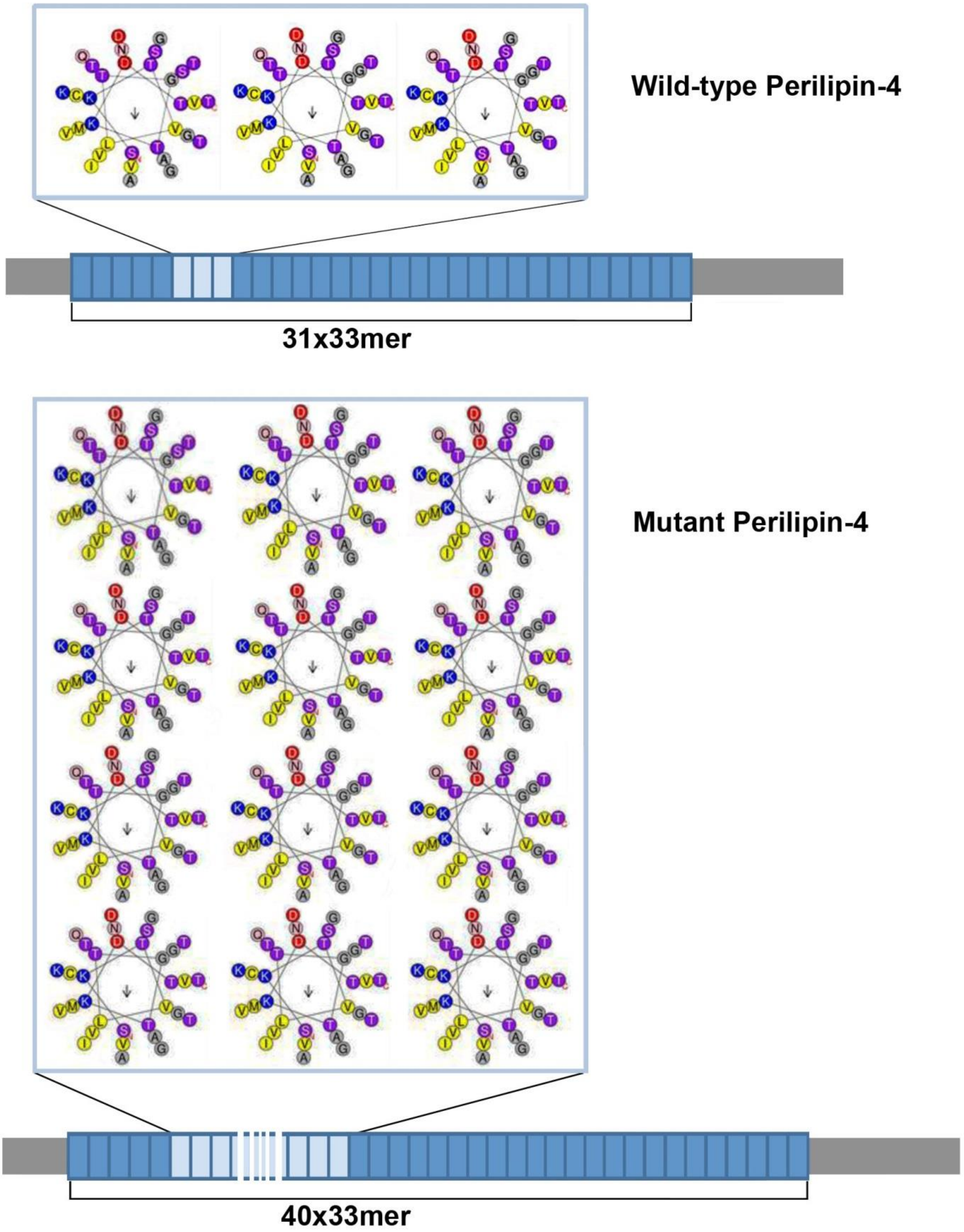

**Supplementary Fig. 7** Perlipin-4 and p62/SQSTM1 co-localization

Immunohistochemistry showing upregulation of p62/SQSTM1 and its co-localization with perilipin-4 in vacuoles and fiber subsarcolemmal regions in a patient, but not in control muscle.

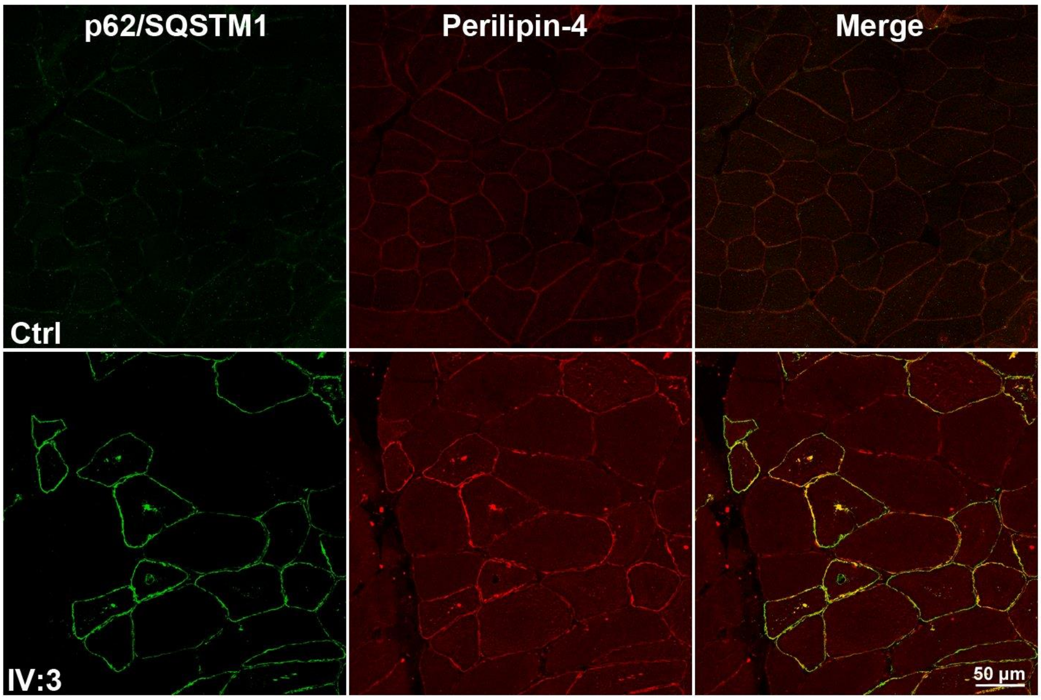


**Supplementary Fig. 8.**Oil Red O staining.

Oil Red O staining, showing similar lipid content in a patient muscle biopsy compared to a control.

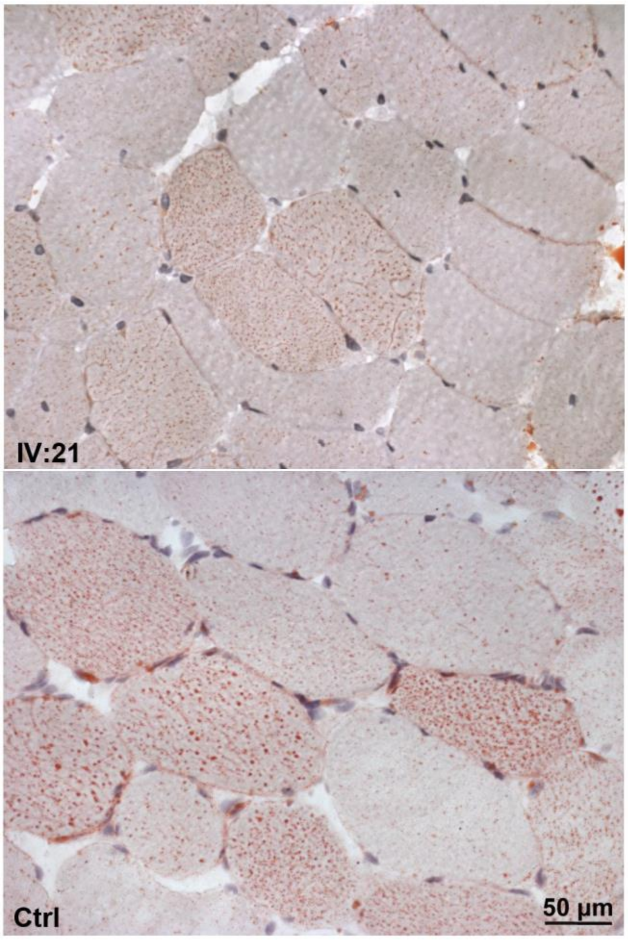

## MATERIALS AND METHODS

**Patients:**

Patients were studied with informed consent to use biological samples for diagnosis and research. Investigations were conducted in accordance with protocols approved by the institutional review boards of the C. Besta Neurological Institute of Milan and The Hospital for Sick Children of Toronto.

In all patients creatine kinase was normal or slightly increased. Electromyography showed myopathic potentials in proximal and distal limb muscles in all affected individuals and frequent repetitive high frequency discharges. Clinical features, initially affecting distal lower limb muscles, progressed over time with weakness of scapular muscles.

Examination of the asymptomatic patient (V:13) at the age of 32 years revealed, on the Medical Research Council (MRC) scale, hand extensor (4/5), neck flexor (4/5) and hallux extensor (4/5) muscle weakness. Electromyography showed positive repetitive complex discharges at needle insertion and myopathic pattern, particularly in tibialis anterior and biceps brachialis muscles. Biopsy of quadriceps muscle showed some centralized nuclei and vacuoles in three myofibers.

**Morphological studies:**

Biopsies of the quadriceps muscles from patients IV:3, IV:10, IV:21, IV:23, IV:24, and V:13 were recently obtained, snap-frozen in isopentane/liquid nitrogen, and maintained in liquid nitrogen. These muscle samples, together with those already available from patients, IV:12 and IV:17, were processed for morphological studies. Transverse cryostat sections were routinely stained for: haematoxylin-eosin, Gomori trichrome, periodic acid-Schiff, Oil Red O, myofibrillar ATPase, acid phosphatase, NADH dehydrogenase, cytochrome C oxidase, succinic dehydrogenase. Immunofluorescence was performed using primary antibodies to: poly-ubiquitinylated proteins (monoclonal PW8810, clone FK2 from Biomol, Enzo Life Sciences, Inc. Farmingdale, USA; 1:100), p62 (guinea pig polyclonal GP62-c, Progene Biotechnik, Heidelberg, Germany; 1:100), p62 (rabbit polyclonal P0067, Merck Sigma-Aldrich, Darmstadt, Germany 1:100) Perilipin-4 (guinea pig polyclonal GP34, Progene Biotechnik, Heidelberg, Germany; 1:100), NBR1 (mouse monoclonal 4BR, Santa Cruz Biotechnology Inc, Santa Cruz CA, USA; 1:100), WDFY3 (mouse monoclonal B-4, Santa Cruz Biotechnology Inc, Santa Cruz CA, USA; 1:100). Cryosections were incubated in the primary antibodies for 2hr, followed by incubation in the secondary antibodies anti-guinea pig, or anti-mouse IgG,Alexa 555 or Alexa 488 conjugated (Invitrogen Life

Technologies, Carlsbad, CA, USA; 1:1500), for 1 hr. Muscle sections were examined under either a Zeiss Axioplan fluorescence microscope (Carl Zeiss AG, Oberkochen, Germany) or a Leica confocal microscope equipped with hybrid and argon lasers (Leica Microsystems, Wetzlar, Germany). Small fragments of muscle tissues, fixed in 4% glutaraldehyde and embedded in Epon 812 resin (Electron Microscopy Sciences, Hatfield PA, USA), were stained with UranyLess (Electron Microscopy Sciences, Hatfield PA, USA) and examined under a FEI Technai electron microscope.

To quantify percentages of fibers positive to perilipin-4 counts were done on pictures of muscle biopsies (at least 3 fields), taken at 20X magnification. To quantify colocalization of perilipin-4/NBR1, perilipin-4/FK2, and perilipin-4/p62, the Fiji plugin JaCoP (JustanotherColocalizationPlugin) analysis was used [2]. The measurement of perilipin-4 overlapping with NBR1, FK2 and p62 was calculated on the entire field of three different images of muscle tissue from a patient and a control (a VCP-mutated patient with autophagic vacuoles). Thresholds were automatically calculated by the software and not set by the operator to avoid biased data. The co-localization measures were reported as Pearson's $r^2$ units, and comparisons between the two groups were done by two-tailed unpaired t-test.

**Western Blot analysis:**

Muscle tissue fragments were homogenized in a buffer containing 20 mM Tris (pH 7.8), 137 mM NaCl, 2.7 mM KCl, 1 mM MgCl2, 1% Triton X-100, 10% (w/v) glycerol, 1 mM EDTA, 1 mM dithiothreitol supplemented with protease inhibitor cocktail (P8340, Merck Sigma-Aldrich, Darmstadt, Germany) and incubated 1h at 4°C. The samples were then centrifuged at 13,000 rpm for 10 min and the supernatants collected. Protein content was measured with the DC protein assay (Bio-Rad). Samples run on a 7.5% or 4-15% acrylamide gels, were transferred into a nitrocellulose membrane for immunological detection using the following primary antibodies: perilipin-4 (guinea pig polyclonal AP33136SU-N, OriGene, Rockville, USA) and SDH70 (anti-complex II 70 kDa Fp subunit monoclonal antibody, cod 459200, Invitrogen, Thermo Fisher Scientific, Carlsbad, California, USA) as loading control.

**Linkage analysis:**

A combination of Illumina chip arrays was used to generate data for whole genome linkage analysis. In detail, SNPs from Illumina Omni1 chip formerly used for CNV analysis on two patients (IV:10 and IV:17) were combined with SNPs from Illumina Omni2.5 array run for patients III:18, IV:3, IV:23 and V:13. Linkage dysequilibrium-based SNP pruning on communal

SNPs was then performed using PLINK software [8] and MERLIN software (version 1.1.2) [1] was used for parametric linkage analysis with parameters considering a rare, dominant and fully penetrant disorder (0.0001, 1.0, 1.0). To further define the exact boundaries of the haplotype shared among the affected members of the family, we combined our previous microsatellite analysis results with the SNPs array data.

**Genome sequencing and variants filtering:**

Library preparation and subsequent sequencing were performed at The Centre for Applied Genomics in Toronto, following Illumina TruSeq Nano DNA library preparation protocol. In brief, indexed TruSeq Illumina adapters with overhang-T were added to the A-tailed fragments of DNA obtained with a Covaris LE220 instrument. Six cycles of PCR were performed for the enrichment of the libraries and were validated with Bioanalyzer DNA High Sensitivity chip and quantified by qPCR using Kapa Library Quantification Illumina/ABI Prism Kit protocol (KAPA Biosystems). The sequencing of the equimolar pooled libraries was performed on an Illumina HiSeq X platform following Illumina's protocol, generating paired-end reads of 150-bases in length. The variants obtained were filtered based on the linkage analysis results, keeping only the heterozygous ones present in both affected patients and not shared by the unaffected siblings. Moreover, the remaining variants were filtered for frequency, excluding those with a minor allele frequency >0.01 based on 1000genomes, ExAc, ESP and ClinVar databases. Genome coordinates refer to reference genome assembly GRCh37 hg19.

**RNAseq:**

For the analysis, two samples were prepared with the NEBNext Ultra Directional RNA library prep kit for Illumina with poly(A) enrichment. After verifying RNA quality and quantity, 500ng of total RNA were enriched for poly-A mRNA, retrotranscribed to double stranded cDNA and modified for Illumina adapters ligation. Amplification of the library fragments was executed adding a different barcode adapter at each step, to allow for multiplex sequencing. Libraries pooled in equimolar quantities were pair-end sequenced on an Illumina HiSeq 2500 platform following Illumina's protocol to generate paired-end reads of 126-bases in length.

**Laser microdissection, protein digestion and mass spectrometry:**

Frozen sections from two affected samples (IV:12 and IV:21) were collected on polyethylene terephthalate membranes (Leica Microsystems) and stained with hematoxylin and eosin (H&E). By laser microdissection using LMD 6500 (Leica Microsystems), different tissue's areas (a total

of 250,000 mm2 areas each) were collected into tubes containing 40 µl of formic acid. The areas were selected as following: rimmed vacuoles (defined as VAC), an equivalent portion of the sarcolemma of the vacuolated fibers (defined as PCI, patient control I), and an equivalent portion of the sarcolemma of healthy-looking fibers (defined as PCII, patient control II) (See Supplementary Fig. 1a). A tryptic in solution-digestion was then carried out as described, followed by nano high-performance liquid chromatography-mass spectrometry (nanoHPLC-ESI-MS/MS) [5].

**Relative protein quantification:**

Using the Mascot search engine (Matrixscience, London, UK) with parameters as previously reported [7], the data obtained by ESI-MS/MS analysis mass spectrometry were searched against the entire Uniprot/Swissprot (release 2014/10, 546,790 entries) database. Evaluation of the quality of identified peptide spectrum matches and protein quantification were performed as reported [7], and the obtained data were normalized by expressing in percentage the proportion of spectral counts for each protein versus the total sum of spectral counts in the individual samples. For each protein, the ratios between the averaged proportions in rimmed vacuoles and control samples were calculated and statistical significance was evaluated with a two-tailed unpaired t-test (equal variances assumed).

**Exon 3 length evaluation by PCR amplification:**

100ng of genomic DNA from all the available family members, were amplified using the following primers 3F-ACCACTCGGTCTGCACTTAand 3R-CCTCCAACTCATTCTGCAGC. In detail, a 20µl of the reaction mixture included: 1x Buffer Phusion GC (F519L, Thermo Fisher Scientific, Carlsbad, California, USA), 200µM each dNTPs (dNTPs mix, 11581295001, Roche Merck, Darmstadt, Germany), 0.5µM each forward and reverse primers, 3% DMSO, 0.4µl Phire Hot Start II DNA Polymerase (F122S, Thermo Fisher Scientific, Carlsbad, CA, USA). The amplified products were subsequently run on a 1% agarose gel and the presence of a single (wt) or two (wt and mut) bands was observed under a UV transilluminator equipped with a Kodak EDAS 290 camera (Eastman Kodak Company, Rochester NY, USA).

**Oxford Nanopore long read sequencing:**

Nanopore sequencing was performed on 500ng of linearized plasmids (pCMV6-AN-GFP) containing either the wild type or the mutant PLIN4 allele of patient cDNA retrotranscribed from

total muscle RNA extract. Both samples were multiplexed with the Native Barcoding kit (EXP-NBD103, Oxford Nanopore Technologies) then prepared for sequencing with the 1D Ligation Sequencing Kit (SQK-LSK108, Oxford Nanopore Technologies) using the manufacturer's recommendations. Base calling and demultiplexing were performed offline with Guppy version 3.2.4 (Oxford Nanopore), using optional parameters '--chunks_per_runner 1500' in high accuracy mode for base calling and '--trim_barcodes --num_extra_bases_trim 30 --barcode_kits "EXP-NBD103"' for demultiplexing. Demultiplexed reads were then filtered for full-length plasmids within an expected size range of 10-12.5 kilobases, resulting in 1121 (wt) and 1164 (mut) full-length reads, which were then subjected to de novo assembly using Canu version 1.8 [5] using options 'genomeSize=12k corMhapOptions="--threshold 0.8 --ordered-sketch-size 1000 --ordered-kmer-size 14" corrected Error Rate=0.105'. The contig assembled from the most reads was then aligned to the human reference genome (GRCh38/hg38) using minimap2 version 2.17 [6] with options '-x splice' to extract the inserted cDNA sequence. The extracted sequence was then polished using Medaka version 10.1 (Oxford Nanopore Technologies) using parameters '-m r941_min_high'.

## References


1. Abecasis GR, Cherny SS, Cookson WO, Cardon LR (2002) Merlin-rapid analysis of dense genetic maps using sparse gene flow trees. Nat Genet 30:97-101. https://doi:10.1038/ng786.
2. Bolte S, Cordelières FP (2006) A guided tour into subcellular colocalization analysis in light microscopy. J Microsc. 224(Pt 3):213-32. https://doi:10.1111/j.1365-2818.2006.01706.x.
3. Di Blasi C, Moghadaszadeh B, Ciano C, Negri T, Giavazzi A, Cornelio F et al. (2004) Abnormal lysosomal and ubiquitin-proteasome pathways in 19p13.3 distal myopathy. Ann Neurol 56:133-8. https://doi:10.1002/ana.20158.
4. Gautier R, Douguet D, Antonny B, Drin G (2008) HELIQUEST: a web server to screen sequences with specific α-helical properties. Bioinformatics 24:2101-2102. https://doi:10.1093/bioinformatics/btn392.
5. Koren S, Walenz BP, Berlin K, Miller JR, Bergman NH, Phillippy AM (2017)Canu: scalable and accurate long-read assembly via adaptive k-mer weighting and repeat separation. Genome Res 27:722-736. https://doi:10.1101/gr.215087.116.
6. Li H (2018) Minimap2: pairwise alignment for nucleotide sequences. Bioinformatics. 34:3094-3100. https://doi:10.1093/bioinformatics/bty191.
7. Maerkens A, Olivé M, Schreiner A, Feldkirchner S, Schessl J, Uszkoreit J, Barkovits K, Güttsches AK, Theis V, Eisenacher M, Tegenthoff M, Goldfarb LG, Schröder R, Schoser B, van der Ven PF, Fürst DO, Vorgerd M, Marcus K, Kley RA (2016) New insights into the protein aggregation pathology in myotilinopathy by combined proteomic and

immunolocalization analyses. Acta Neuropathol Commun 4:8. https://doi:10.1186/s40478-016-0280-0.

8. Purcell S, Neale B, Todd-Brown K, Thomas L, Ferreira MA, Bender D, Maller J, Sklar P, de Bakker PI, Daly MJ, Sham PC (2007) PLINK: a tool set for whole-genome association and population-based linkage analyses. Am J Hum Genet 81:559-575. https://doi:10.1086/519795.
9. Robinson JT, Thorvaldsdóttir H, Winckler W, Guttman M, Lander ES, Getz G, Mesirov JP. (2011) Integrative Genomics Viewer. Nature Biotechnology 29:24-26. https://doi:10.1038/nbt.1754.
